# Data on the prevalence of addiction to the Internet among individuals with a history of drug abuse

**DOI:** 10.1016/j.dib.2018.09.052

**Published:** 2018-09-24

**Authors:** Katayoon Razjouyan, Peiman Hamzenejhad, Mojgan Khademi, Fariba Arabgol

**Affiliations:** Child and Adolescent Psychiatrist in Shahid Beheshti University of Medical Sciences, Behavioral Sciences Research Center, Imam Hossein Hospital, Tehran, Iran

**Keywords:** Internet addiction, Drug use, Iran, Kerman

## Abstract

The purpose of this data article is to present the prevalence of Internet addiction in people with a history of substance abuse disorder in Kerman during 2016–2017. For this purpose, 223 people with the history of substance abuse disorder in three cities of Kerman province completed the demographic form and Young׳s Internet addiction questionnaire. Demographic characteristics of participants was analyzed and presented here. Young׳s Addiction Commitment Questionnaire contains 20 questions, each with a score of 1 to 5. The history of participants about the drug abuse was investigated. In addition, the prevalence of Internet addiction among the participants was evaluated. The internet addiction is more common in people with a history of drug abuse which may be a behavioral substitute for drug addiction.

**Specifications table**

**Table:** 

Subject area	Social sciences
More specific subject area	Behavioral psychiatry
Type of data	Tables
How data was acquired	223 people with a history of substance abuse disorder in three cities of Kerman province completed a demographic form and Young׳s Internet addiction questionnaire, containing 20 questions.
Data format	Raw and analyzed
Experimental factors	For all questions in the questionnaire, the scoring was done by a Likert Scale of 1–5.
Experimental features	Based on the Internet addiction, the participants were classified into three groups: minor addiction, moderate addiction, and severe addiction.
Data source location	Kerman, Kerman province, Iran.
Data accessibility	Data are included in this article
Related research article	J.-Y. Yen, C.-F. Yen, C.-C. Chen, S.-H. Chen, C.-H. Ko, Family factors of Internet addiction and substance use experience in Taiwanese adolescents, CyberPsychology & Behavior. 10 (2007) 323–329 [1].

**Value of the data**

• This data can be used for authorities to be aware of the role of drug abuse on Internet addiction
• The data can be interesting for psychiatrists studying on Internet addiction or drug abuse.
• This data can be interesting for other researcher from other countries to investigate the prevalence of Internet addiction among people with drug abuse.

## 1 Data

Of the 223 participants, 215 (96.4%) were male and 8 (3.6%) were female. Most were married (61%) and graduate students (38.6%). The mean age of these people is about 35.5 years and the mean age of the first use of drugs is 18 years. The minimum age for starting the drug in these people is 6 years and the highest starting age is 40 years. 173 subjects (77.6%) had a history of opium addiction; alcohol addiction (84 subjects, 37.7%) and methadone (64 subjects, 28.7%) were in the following categories in terms of addiction type. 18.8% of them had a history of physical illness and 14% had a history of psychological disorders. Of the 223 patients, 26 (11.7%) had no dependence on the Internet, 123 (55.2%) had minor addiction, 57 (25.6%) had moderate addiction and 17 (7.6%) had severe addiction (Table 1).

**Table 1:** The frequency and severity of addition among respondents.

**Severity of Internet addition**		**Frequency**	**Percentage (%)**
Internet addiction	No addiction	26	11.7
	Minor	123	55.2
	Moderate	57	25.6
	Severe	17	7.6

Table 2 shows the relationship between the history of drug addiction and the severity of Internet addiction in the subjects under review.

**Table 2:** The relationship between the history of drug addiction, on the basis of the type of drug, and the severity of Internet dependency.

**History**	**Yes/No**	**The intensity of Internet addiction**			
		**No dependence**	**Minor dependence**	**Moderate dependence**	**Severe dependence**
		**Frequency (percent)**	**Frequency (percent)**	**Frequency (percent)**	**Frequency (percent)**
History of cannabis addiction	No	20(13.7%)	79(54.1%)	39(26.7%)	8(5.5%)
	Yes	6(7.8%)	44(57.1%)	18(23.4%)	9(11.7%)
History of opium addiction	No	8(16.0%)	25(50.0%)	11(22.0%)	6(12.0%)
	Yes	18(10.4%)	98(56.6%)	46(26.6%)	11(6.4%)
History of heroin addiction	No	14(8.7%)	89(55.3%)	44(27.3%)	14(8.7%)
	Yes	12(19.4%)	34(54.8%)	13(21.0%)	3(4.8%)
History of crack addiction	No	22(10.8%)	112(55.2%)	54(26.6%)	15(7.4%)
	Yes	4(20.0%)	11(55.0%)	3(15.0%)	2(10.0%)
History of morphine addiction	No	23(11.2%)	112(54.6%)	54(26.3%)	16(7.8%)
	Yes	23(11.2%)	112(54.6%)	54(26.3%)	16(7.8%)
History of crystal addiction	No	18(9.8%)	107(58.5%)	44(24.0%)	14(7.7%)
	Yes	8(20.0%)	16(40.0%)	13(32.5%)	3(7.5%)
History of alcohol addiction	No	18(12.9%)	81(58.3%)	33(23.7%)	7(5.0%)
	Yes	8(9.5%)	42(50.0%)	24(28.6%)	10(11.9%)
History of sleep drug addiction	No	24(13.1%)	97(53.0%)	48(26.2%)	14(7.7%)
	Yes	2(5.0%)	26(65.0%)	9(22.5%)	3(7.5%)
Methadone addiction history	No	19(11.9%)	87(54.7%)	41(25.8%)	12(7.5%)
	Yes	7(10.9%)	36(56.3%)	16(25.0%)	5(7.8%)

## 2 Experimental design, materials and methods

Two hundred twenty-three (223) people with history of substance abuse disorder in three cities of Kerman province completed demographic form and Young׳s Internet addiction questionnaire. Sampling was done in an accessible way among subjects who have been taking at least one month since the last substance abuse. Before the questionnaires were completed, we explained to participants about the purpose of this study and gave them a brief guidance on how to complete the questionnaires. Demographic data included age, sex, education, employment, marital status, and the duration of drug use, history of substance or substances that contribute to addiction, history of substances dependence, duration of the avoidance period, type of health plan, history of physical illness, and the history of mental illnesses.

Young׳s Questionnaire and Internet addiction Criterion consists of 20 questions. The reliability and validity of which has been proven by Alavi et al. study at Isfahan University (Cronbach׳s *α* = 0.88 and *r* = 0.82) [2]. The lowest score is 20 and the highest score is 100. The classification of the intensity of the addiction is like the following:

● 21–49 minor addictions.
● 50–79 moderate addiction.
● 80–100 severe addictions.

Data from questionnaires were extracted by two independent investigators. The data were entered to an Excel spreadsheet (Microsoft Office 2013). The demographic characteristics of the participants were investigated and presented in this data article. The score of addiction was calculated for each person. Based on this score, the participants were classified to different groups. The number of relative frequency of people in each group was calculated and presented in this data article. Finally, the participants were classified based on the type of drug that they have used before, and the number and relative frequency of participants in each group were calculated. Data analysis was performed using SPSS 22 [3], [4]. The result showed the internet addiction is more common in people with a history of drug abuse which may be a behavioral substitute for drug addiction.
